# Ultrasound Used for Diagnostic Imaging Facilitates Dendritic Branching of Developing Neurons in the Mouse Cortex

**DOI:** 10.3389/fnins.2022.803356

**Published:** 2022-03-17

**Authors:** Tamas Papp, Zsuzsanna Ferenczi, Bernadette Szilagyi, Matyas Petro, Angelika Varga, Eva Kókai, Ervin Berenyi, Gabor Olah, Gabor Halmos, Peter Szucs, Zoltan Meszar

**Affiliations:** ^1^Department of Medical Imaging, University of Debrecen, Debrecen, Hungary; ^2^Department of Anatomy, Histology and Embryology, University of Debrecen, Debrecen, Hungary; ^3^Department of Biopharmacy, University of Debrecen, Debrecen, Hungary; ^4^MTA-Debreceni Egyetem, Neuroscience Research Group, Debrecen, Hungary

**Keywords:** ultrasound, neuron, neurogenesis, dendrite, fetus

## Abstract

Neuronal differentiation and synaptogenesis are regulated by precise orchestration of intrinsic and extrinsic chemical and mechanical factors throughout all developmental steps critical for the assembly of neurons into functional circuits. While ultrasound is known to alter neuronal migration and activity acutely, its chronic effect on neuronal behavior or morphology is not well characterized. Furthermore, higher-frequency (3–5 MHz) ultrasound (HFU) is extensively used in gynecological practice for imaging, and while it has not been shown harmful for the developing brain, it might be associated with mild alterations that may have functional consequences. To shed light on the neurobiological effects of HFU on the developing brain, we examined cortical pyramidal cell morphology in a transgenic mouse model, following a single and short dose of high-frequency ultrasound. Layer V neurons in the retrosplenial cortex of mouse embryos were labeled with green and red fluorescent proteins by *in utero* electroporation at the time of their appearance (E14.5). At the time of their presumptive arrival to layer V (E18.5), HFU stimulation was performed with parameters matched to those used in human prenatal examinations. On the third postnatal day (P3), basic morphometric analyses were performed on labeled neurons reconstructed with Neurolucida. Low-intensity HFU-treated cells showed significantly increased dendritic branching compared to control (non-stimulated) neurons and showed elevated c-fos immunoreactivity. Labeled neurons were immunopositive for the mechanosensitive receptor TRPC4 at E18.5, suggesting the role of this receptor and the associated signaling pathways in the effects of HFU stimulation.

## Introduction

Ultrasound (US) is a mechanical pulse wave with a frequency range above the upper perceptual limit of human hearing (>20 kHz). Its physical properties allow deep penetration into solid structures including soft and hard tissues of the body, thus making it suitable for medical diagnostic imaging, in a frequency range between 1 and 15 MHz.

In gynecological practice, US examination during pregnancy is crucial to following the development of the fetus and to reveal potential morphological abnormalities. For examination of the fetus, the convex abdominal and vaginal head is used (frequency is 3–5 MHz) with mechanical index (MI) < 1 and thermal index (TI) < 1 (Kurjak and Chervenak, 2004; Sheiner et al., 2007; Maeda, 2014; Callen et al., 2017). The MI and TI are important descriptors of US, while TI indicates the possible elevation of temperature in the tissue, and MI indicates the bioeffect of the pressure of US waves (Jago, 1995; Lee, 1998; Maeda, 2014). The average time of US examination is about 10 min, but nowadays 3D, 4D “baby movies” can be as long as 30–40 min (Sheiner et al., 2007; Sasaki et al., 2010; Callen et al., 2017). While diagnostic fetal US does not cause any physical and mental developmental disorder at a population level, it might be related to several minor alterations, such as increased frequency of left-handedness, elevated body weight, and higher incidence of delayed speech (Kieler et al., 2002; Torloni et al., 2009; Smarr et al., 2018). The obstetrical US during the second and third trimesters of pregnancy mechanically stimulates developing neurons in the cortex and the hippocampus, as this interval includes the axo-dendritic growth and circuit assembly, particularly in the limbic system, involved in emotion, motivation, learning, and memory (Rakic and Yakovlev, 1968; Clancy et al., 2001).

Neurogenesis has a very precise spatial and temporal schedule (Cooper, 2008). The excitatory neurons derive from the ventricular zone of the cortical plate and build the cerebral cortex in an inside-out manner; earlier-born neurons settle at the deeper laminae while younger neurons populate laminae close to the pial surface by migrating through the early born layer. Progenitor cells of the cortical inhibitory neurons are found in the medial ganglionic eminence, and postmitotic neurons migrate toward the cortical plate under the pia mater or in the subventricular zone (Stiles and Jernigan, 2010). US stimulates the proliferation of neuroprogenitor cells, and it can constrain the migration of some cortical pyramidal neurons in the developing cerebral cortex (Ang et al., 2006). Low-intensity US treatment also facilitates the survival and differentiation of neuronal stem cells *in vitro* (Lee et al., 2014) and *in vivo* (Tufail et al., 2011). Following migration and lamination, appropriate dendritic growth and branching are essential for the proper circuit formation and correct function of neurons. Dendritic arborization of neurons determines their synaptic input and membrane characteristics, and coordinated dendritic growth is largely determined by environmental as well as intrinsic factors. The brain-derived neurotrophic factor (BDNF) as one of these intrinsic factors has a pivotal role during dendritic arborization, promoting the elaboration of the dendritic tree, while it is not required for the proliferation, survival, or migration (Gorski et al., 2003; Jan and Jan, 2010). US as an extrinsic factor also caused rapid and transient changes of neurite growth in neuronal cell culture (Hu et al., 2013), through mechanosensitive receptors and ion channels. The transient receptor potential channels (TRPCs) are widely expressed in developing murine brains including the cortical plate (Fowler et al., 2007; Zechel et al., 2007). Among them, TRPC4 can be activated by US with a frequency above 2 MHz (Ibsen et al., 2015), which raised the possible involvement of TRPC4 in US-mediated signaling in developing neurons. TRPC4 is a non-selective cation channel located mostly in the membrane of the endoplasmic reticulum and increases the cytoplasmic Ca^2+^ level upon activation (Sun et al., 2014). Local intracellular Ca^2+^ signaling influences dendritic growth and branching for example through CaM kinase and CREB (Redmond et al., 2002).

Thus, in this study, we demonstrated the expression of the mechanosensitive TRPC4 receptor in developing cortical neurons. We characterized the effect of the low-intensity and high-frequency US (HFU) used in obstetrical practice on developing pyramidal neurons in the limbic retrosplenial cortex. Our results demonstrate that a single and short-term US exposure of developing mouse fetuses in the mother leads to a significant increase in the number of dendrites of cortical pyramidal cells of the offspring.

## Materials and Methods

### Animals

For *in utero* electroporation, time mated pregnant CD1 (ICR, Charles River, Germany) mice were used. Altogether, 5 pregnant mice with 24 electroporated embryos (survived 20, selected 10 for morphometry and immunohistochemistry) were subjected to US stimulation and 5 pregnant mice with 21 electroporated embryos (survived 18, selected 7 for morphometry and immunohistochemistry) were used as control. Additional 3 US-stimulated and 3 control pregnant mice were sacrificed for collecting 9 US-stimulated and 9 control pups (3 from each pregnant mouse). For timing of the pregnancy, the copulation plugs were observed in the morning which was referred to as embryonic day 0.5 (E0.5) of the zygotes. Animals were handled and housed according to the guidelines of the Animal Care Committee of the University of Debrecen, Debrecen, Hungary (Approval No.: 10/2016/DE-MAB), and the national laws and regulations of the European Union (Directive 2010/63/EU).

### Plasmids

An expression vector coding the EGFP sequence was designed and constructed by our lab (pCALN-loxp-GFP-loxp-CS3) that expressed a cre-recombinase deletable GFP under a CAG promoter. Since we did not use cre deletion in this vector in this study, we used this vector as a ubiquitous GFP expression vector. The pCAG-cre that contains cre-recombinase was provided by Dr. J. Miyazaki (Division of Stem Cell Regulation Research, Osaka University Medical School, Osaka, Japan). The Cytbow vector, a multicistronic piggyback plasmid, expresses three fluorescent reporters (tdTomato, turquoise, and EYFP) regulated by three different lox sequences (Loulier et al., 2014), which was provided by Karine Louiller (Vision Institute—INSERM, Paris, France). For activating the Cytbow expression, we used the pCAG-Cre vector.

### *In utero* Electroporation

Neuronal morphology changes after US exposure were assessed on GFP and the Cytbow-labeled layer V pyramidal neurons in the murine retrosplenial cortex. To be able to focus on changes caused by US and to reduce the effect of the inherent variability of pyramidal cell morphology, we compared cells that were born in the same time interval, migrated into the same layer, and thus were likely in the same differentiation state. This could be best achieved by *in utero* electroporation of the plasmids with fluorescent reporter genes since the electroporated plasmid DNA provides a stable gene expression only in those of postmitotic neurons which left the cell cycle at the same time as the electroporation. For labeling of layer V pyramidal neurons in the retrosplenial cortex, we performed *in utero* electroporation at E14.5 using the procedure described by Navarro-Quiroga et al. (2007). Briefly, pregnant mice at E14.5 were anesthetized deeply with sodium pentobarbital (50 mg/kg); their uterine horns were exposed through an incision on the abdominal wall. Embryos were identified, and the plasmid solution was injected into their right lateral ventricle. The injected embryos were then electroporated by an Electro Square Porator (BTX, ECM830), holding the embryos in the uterus slightly obliquely (15°–30°) to the anode, using the interaural line as a reference plane of 0°. The electric pulses were delivered by forceps-type electrodes (BEX, LF650P5), as follows: 5 pulses of 40 V for 50 ms in every second. The distance between the anode and cathode was set to 6 mm with the calculated electric field 67 V/cm. The uterus was then washed with saline and placed back into the abdominal cavity, which was then closed by two-layer sutures.

### Ultrasound Exposure

Mice that previously underwent *in utero* electroporation were deeply anesthetized with Na-pentobarbital (50 mg/kg), and US stimulus was applied on E18.5 for 10 min. The US device was a GE Logiq V2, the frequency used was constantly 3 MHz, and both mechanical and thermic indexes were kept under 1.0 (MI = 0.9; TIS = 0.8; other parameters: D: 17, DR: 69 dB, AO%: 100). The used abdominal US head was a 4C-RS convex probe (frequency range: 2.0–5.0 MHz, number of elements: 128, convex radius: 60 mmR, FOV: 55°, footprint: 18.3 × 66.2 mm, B-mode imaging frequency: 2.0, 3.0, 4.0, 5.0 MHz, harmonic imaging frequency: 3.0, 4.0, 5.0 MHz). The transducer was covered by AquaUltra Basic US gel (Ultragel, Budapest, Hungary), and the transducer was gently placed to the belly of the mouse (in case of embryonic treatment) or directly to the skull of the animals (after birth). The focus of US was in the midline of the pregnant mice during the E18.5 treatment (which was from the same distance from the uterine horns); after birth, unfocused US was used related to the small size of the skull. The US probe covered the territory of the abdominal region of the pregnant mice, indicating that all embryos got the same US dose independently from the exact position in the embryos. The calculated average intensity during the exposition was 56 mW/cm^2^, and the calculated peak negative acoustic pressure was 1.557 MPa. In the case of five times repetitive US exposure experiment, the first treatment took place at E18.5 (*in utero*), which was followed by 4 further US stimuli once a week at the first 4 postnatal weeks. Control animals underwent the same manipulations [i.e., the mouse was anesthetized, *in utero* electroporation (except the samples for qRT-PCR)]. The US transducer was pressed to the abdomen as the US-stimulated ones without being subjected to actual US exposure.

### Neuron Reconstruction and Morphometry

We selected layer V pyramidal cells in the retrosplenial cortex for 3D reconstruction with Neurolucida (MBF Bioscience, Williston, VT, United States) from confocal image stacks. The confocal images were collected at 0.5-μm steps with a × 40 (Olympus, UplanFLN, NA:1.30) oil immersion objective on an FV3000 microscope (Olympus, Tokyo, Japan). Manually guided 3D reconstructions were performed on GFP- and Brainbow-labeled neurons (20–20 GFP-labeled and 15–15 Brainbow pyramidal cells) from control and US-treated animals. The contours of the cell bodies were carefully drawn at all Z levels in the image stacks. Neuronal dendrites were reconstructed faithfully, taking note of the process’s diameter at every single reconstructed point. In the retrosplenial cortex, the deepest layer V pyramidal cells were selected to ensure that only cells in layer V are compared. Data from GFP- and Brainbow-labeled pyramidal cells were pooled as no differences between the pyramidal neurons labeled with the different plasmids have been observed. The morphometric parameters determined from the reconstructions were total number of neurites, total length, numbers of branchpoints (nodes), and dendritic segment length.

### Immunohistochemistry

Immunofluorescent labeling was used for gaining the fluorescent green GFP signal and detecting the TRPC4, c-Fos, and BDNF in US-treated and control mouse brains. E18.5 embryos and P3 and P30 mouse pups were sacrificed by decapitation, and whole brains were carefully dissected and immersion fixed in 4% paraformaldehyde in 0.1 M phosphate-buffered saline (PBS, pH 7.4) overnight at 4°C. Brain samples were then rinsed with PBS and embedded in 4% agarose, and 100-micron-thick free-floating coronal sections were made with a vibratome (Leica, Wetzlar, Germany). Sections were incubated in primary (2 days at 4°C) and secondary (overnight at 4°C) antibody mixtures. All antibodies were diluted in PBS (pH 7.4) supplemented with 0.3 M NaCl and 0.3% Triton X-100. Three 15-min washes in PBS were performed after incubation.

Details of the primary antibodies applied in this study are listed in Table 1. Species-specific secondary antibodies were raised in donkey or goat and conjugated to Alexa Fluor-488, 555, and 647 (Invitrogen, Carlsbad, CA, United States). At the end of the protocol, the sections were incubated with cell nucleus-specific DAPI (Sigma, D9542, 100 ng/ml) for 2 h at RT to help determine layer boundaries. Sections were mounted in Hydromount medium (National Diagnostics Atlanta, GA, United States) and confocal images were obtained with an Olympus FV3000 confocal system.

**TABLE 1:** List of antibodies used in this study, related to section “Materials and Methods.”

Antibody	Specificity	Host species	Dilution	Source
ab13970	Recombinant full-length protein corresponding to GFP	Chicken	1:2,000	Abcam
SAB2108245	Synthetic peptide directed toward the middle region of human TRPC4	Rabbit	1:100	Sigma-Aldrich
MAB248	Recombinant human BDNF corresponding to the following epitope sequence: Arg128–Arg247	Mouse	1:25	R&D Systems
sc-52	N-terminus of c-Fos of human origin	Rabbit	1:25	Santa Cruz Biotechnology
A-11039	Goat anti-chicken IgY (H + L) secondary antibody, Alexa Fluor 488	Goat	1:500	Invitrogen (Thermo Fisher Scientific)
A-31572	Donkey anti-Rabbit IgG (H + L) highly cross-adsorbed secondary antibody, Alexa Fluor 555	Donkey	1:500	Invitrogen (Thermo Fisher Scientific)
A-11001	Goat anti-mouse IgG (H + L) cross-adsorbed secondary antibody, Alexa Fluor 488	Goat	1:500	Invitrogen (Thermo Fisher Scientific)
A-31571	Donkey anti-mouse IgG (H + L) highly cross-adsorbed secondary antibody, Alexa Fluor 647	Donkey	1:500	Invitrogen (Thermo Fisher Scientific)

### RNA Isolation and cDNA Transcription and qRT-PCR

Whole mouse brain samples (*n* = 9, from 3 litters) were collected 60 min after the US exposure at E18.5. As described above, control animals (*n* = 9, from 3 litters) were handled and treated in the same way as the US-stimulated group but without being subjected to actual US exposure. Total RNA was isolated by TRIzol (Molecular Research Center, Cincinnati, OH, United States) reagent. During the standard isolation, protocol extended with the DNA digestion step, 30 mg of mouse brain tissue was used for isolation and quantified and qualified using a NanoDrop ND-1000 spectrophotometer (Thermo Fisher Scientific, Waltham, MA, United States). 1,000 ng of total RNA was reverse transcribed to cDNA using a Tetro cDNA Synthesis Kit (Bioline, Alvinston, Canada) according to the manufacturer’s instructions. Then, SYBR Green Supermix (Bio-Rad Laboratories, Hercules, CA, United States) with the CFX96 Real-Time System C1000 Thermal Cycler (Bio-Rad Laboratories) was used under the following PCR conditions: 2 min 50°C, 1 cycle 3 min 95°C, 39 cycles 15 s 95°C, 1 min 60°C, and 81 cycles 1 min 55°C–98°C. The following specific primer pairs have been used for BDNF: forward GGCTGACACTTTTGAGCACGTC, reverse CTCCAAAGGCACTTGACTGCTG, HPRT1: forward CAGTCCCAGCGTCGTGATTA, reverse TGGCCTCCCATCT CCTTCAT, GAPDH: forward GTCATCCCAGAGCTGAACGG, reverse TACTTGGCAGGTTTCTCCAGG (OriGene Tech, Rockville, MD, United States). For the quantification of gene expression, the 40-CT method was used. BDNF expression was normalized to the housekeeping genes of HPRT1 and GAPDH. No template controls (NTC, RT-NTC, NRT) were used for detecting the potential contaminants of each step of the reaction.

### Statistical Evaluation

The morphometric and qPCR data between control and US-treated animals were compared using the Mann–Whitney *U*-test using OriginPro (OriginLab Corporation, Northampton, MA, United States).

## Results

### Labeling Pattern of the *in utero* Electroporation at E14.5

**FIGURE 1:**
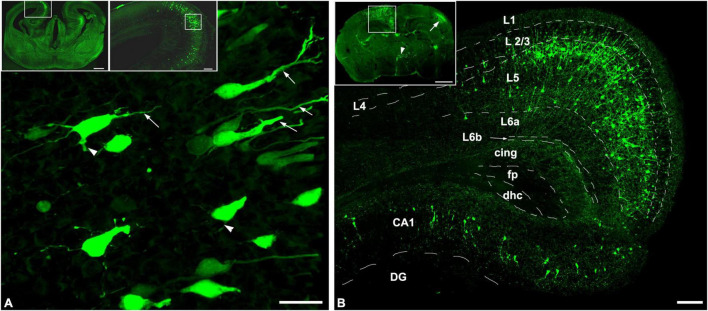
*In utero* electroporation revealed GFP-labeled neurons in the limbic area of the telencephalon. Coronal sections of mouse brain sampled at **(A)** E18.5 and **(B)** P3, after the labeling by utero electroporation was carried out at E14.5. **(A)** The GFP-labeled neurons already populated their presumptive layer in the cortex and the hippocampus which is shown in the upper right panel that is illustrated with the enframed area in the coronal view of the GFP-labeled mouse brain from E18.5 shown in the upper left panel. The labeled neurons showed polarized morphology, with a visible apical dendrite (arrows), and in some cases basal dendritic processes can also be obtained (arrowheads). **(B)** Upper panel shows the GFP (green) labeling pattern includes the retrosplenial cortex and the hippocampus (enframed area), the contralateral-sided somatosensory cortex (arrow), and scattered labeling in the hypothalamus (arrowhead). The retrosplenial cortex and the CA1 region of the hippocampus are shown with higher magnification which is illustrated in the framed area in the upper panel. In the retrosplenial cortex, almost all the GFP-positive pyramidal neurons were found in layer 5 and the hippocampal CA1 pyramidal cells were also labeled. Dashed lines illustrate the borders of regions of the retrosplenial cortex. L1-L6b, Cortical layers; cing, cingulum; fp, posterior forceps of the corpus callosum; dhc, dorsal hippocampal commissure; DG, dentate gyrus; CA1, hippocampal region CA1. Scale bar: 20 μm in **(A)**, 500 μm in upper left panel, 100 μm in upper right panel and **(B)**, and 700 μm in upper in **(B)**.

Since pyramidal neurons populating the presumptive layers of the cerebral cortex and the hippocampus derive from the progenitors of the ventricular zone of the cortical plate, first we examined the distribution pattern of the labeled neurons at the time of the US stimulation (E18.5) and at the time of sampling (P3) revealed by *in utero* electroporation at E14.5 (Figure 1).

Four days after the electroporation, at E18.5, the labeled pyramidal cells were populating their presumptive layer 5 in the retrosplenial cortex and the pyramidal layer in the CA1 region of the hippocampus (Figure 1A, upper panels). The polarized morphology (Hatanaka et al., 2012) was recognizable among the GFP-positive cells. This phenomenon is characterized by the migratory leading process becoming a branchy apical dendrite (Figure 1A). Only some of the GFP-labeled neurons had recognizable basal dendritic processes, indicating that most of the labeled neurons were in the phase of morphological differentiation.

Three days after birth (P3), the GFP positivity in the cerebral cortex includes the frontal, retrosplenial cortices in the injected side and the somatosensory area contralateral to the injected hemisphere (Figure 1B). In the injected hemisphere, most of the GFP-labeled cortical neurons were pyramidal cells and settled in layer 5. Also, mostly pyramidal cells were labeled in the ipsilateral hippocampus which was populating the pyramidal layer of the CA1 region. In cases, the injected DNA was flown into the contralateral hemisphere and the third ventricles during the electroporation, the contralateral hemisphere, and the diencephalon were also labeled by GFP. Mainly the layer 4 interneurons of the somatosensory cortex in the contralateral hemisphere and hypothalamic neurons were labeled due to the transfection of the neural progenitor cells of the medial and lateral ganglionic eminences at E14.5. It is important to underline that the GFP-labeled neurons were born after E14.5 in a close interval and they found their final presumptive layer destinations resulting in an identifiable cortical layer by the age P3. Besides the cortical layers, the pyramidal cell morphology could also be obtained with the two characteristic dendritic domains. This includes the long and branching apical dendrites terminating in layer I and basal dendrites. Subclasses of the layer 5 pyramidal cells were not identifiable at this young age, however.

### Ultrasound Stimulus Increases the Numbers of Basal Dendrites of Layer 5 Pyramidal Cells

**FIGURE 2:**
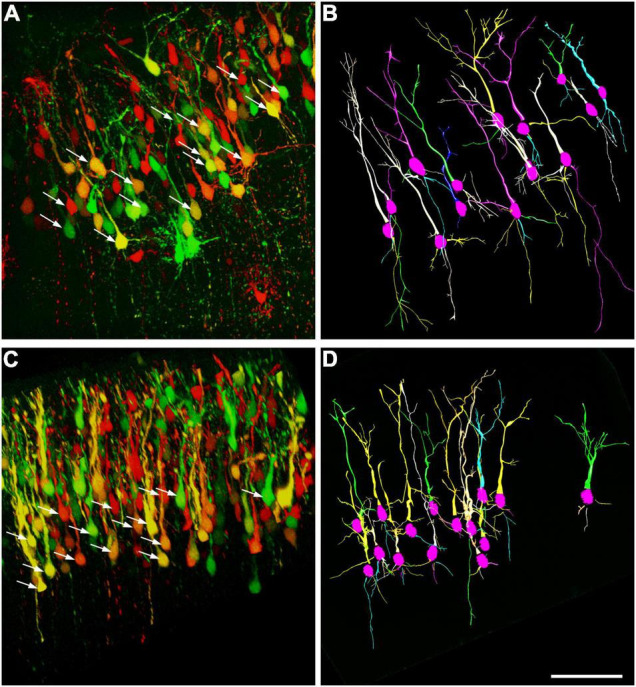
Computer-aided reconstruction of pyramidal cells in the retrosplenial cortex. Maximum intensity projection of 200-μm confocal optical sections forms the frontal cortex where the neurons labeled with Brainbow vector at E14.5 by *in utero* electroporation. Samples were taken on the third day of postnatal stages from a non-treated control **(A)** and a mouse stimulated by a single dose of ultrasound (US) at E18.5 age of fetuses **(C)**. Arrows show those of pyramidal neurons chosen randomly from layer 5 which were analyzed by Neurolucida. The reconstructed pyramidal cells from the control **(B)** and US-stimulated samples **(D)** are illustrated (15–15 pyramidal cells). Scale bar: 200 μm.

**FIGURE 3:**
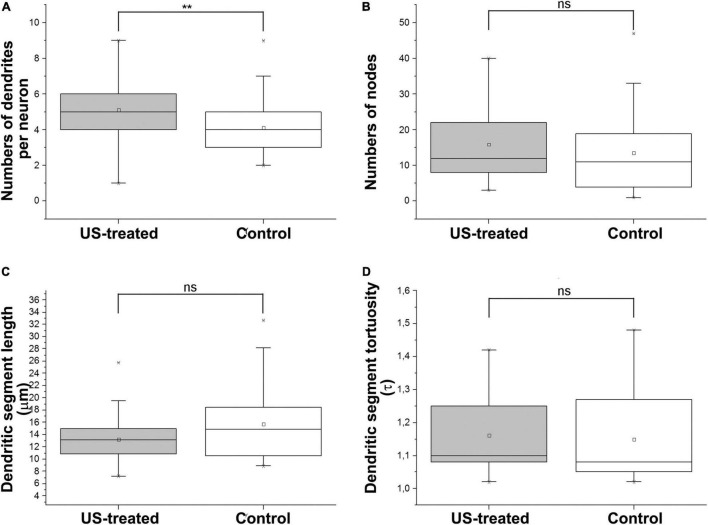
Ultrasound stimulus increases the number of dendrites. Morphometric analyses after reconstruction of the neurons in P3 indicate that a single short-term intrauterine ultrasound (3 MHz) stimulus at E18.5 (US-treated) increased the numbers of dendrites by approx. 1–2 compared to the non-stimulated (Control) group **(A)**. The dendritic morphology was not changed significantly between the US-treated and Control groups. Only a slight and not significant gain was measured in the numbers of nodes **(B)** and the length of the internodal dendritic segments **(C)**, and their tortuosity **(D)** was not changed in the US stimulus. **p < 0.01, ns: not significant (both Mann–Whitney). Boxes are indicating the middle 50% of the data with the median. Bars are indicating the upper and lower 25–25% of data while the asterisk labels are the outlier data. The means are indicated as small center squares in the boxes.

**FIGURE 4:**
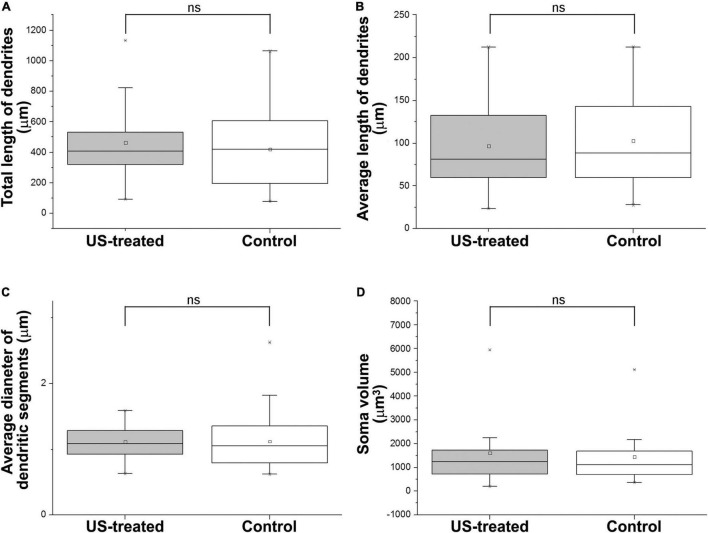
Ultrasound stimulus does not cause any changes in dendrite morphology and soma size. US treatment at E18.5 (at that time the GFP-labeled neurons were 4 days old) was ineffective to alter the length of the dendrites **(A,B)** of the GFP-labeled pyramidal cells at P3. The thickness of the dendrites **(C)** and the size of the soma **(D)** were also the same between the US-treated and the non-treated control groups. ns: not significant (Mann–Whitney). Boxes indicate the middle 50% of the data with the median. Bars indicate the upper and lower 25–25% of data while the asterisk labels are the outlier data. The means are indicated as small center squares in the boxes.

A similar labeling pattern was observed among the pyramidal cells in the case that we used a Brainbow vector for labeling instead of GFP (Figure 2). Since the density of the labeled layer 5 retrosplenial pyramidal cells was high, the difficulty of reconstruction of single cells and processes could be overcome by multicolor fluorescent imaging due to the Brainbow system. Two-dimensional views of the randomly selected reconstructed cells from layer 5 shared very similar morphological properties according to the branching tree of the apical and basal dendrites. A single US stimulation did not cause dramatic changes in pyramidal cell morphology (Figures 2–4). The marked difference could only be registered in the numbers of dendrites (Figure 3A). Since all these reconstructed pyramidal neurons have one apical dendrite, the US stimulation elevated the numbers of basal dendrites by 1–2. The branching tree analyses revealed that the US treatment did not cause any dramatic effects in the branching numbers (Figures 3B,C), and the shape of the dendrites was also like the non-stimulated control (Figure 3D). The Brainbow vector-labeled pyramidal cells were also in layer 5 at P3. The different colors of the cells indicated the cell lines, which gave the most comparable neuron population for the statistics (Figure 2A box). The arborization of the neurons was also well developed. Figure 2B box shows some reconstructed neurons in 3D. To demonstrate that prenatal US exposure affected neuronal morphology, we manually reconstructed the layer V-labeled pyramidal cells with Neurolucida software (MBF Bioscience). No change was observed in the average segment tortuosity, average segment length, terminal distance, number of nodes, and dendrite length of treated and control pyramidal neurons. The only significant change was the elevated number of dendrites in the US-stimulated group.

### Rapid Activation of Neurons After a Single Ultrasound Stimulus

Activation of neurons in the central nervous system can be indicated by their general increase in expression of early response genes particularly c-Fos (Sheng and Greenberg, 1990). Since c-Fos level was elevated by 2–3-fold upon US stimulation in a frequency-dependent manner (Miller et al., 2017), we hypothesize that a higher relative expression of c-Fos increased the expression of downstream morphogenetic molecules like BDNF (Dong et al., 2006), which could be facilitating the outgrowth of the dendrites of maturating neurons in the retrosplenial cortex similarly to the visual cortex described by McAllister et al. (1995).

**FIGURE 5:**
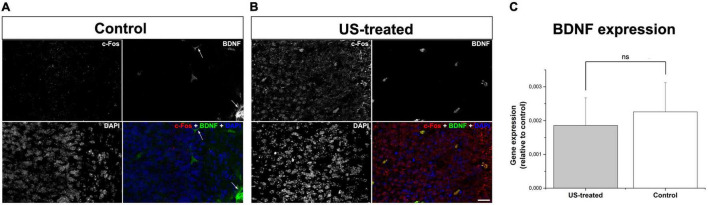
A single stimulation with ultrasound activated neurons but did not increase BDNF expression. Confocal images show immunofluorescent staining of c-Fos (red) and BDNF (green) in the retrosplenial cortex **(A,B)**. Immunoreactivity of c-Fos could not be observed in the non-treated control **(A)** which was markedly increased upon a single US stimulus **(B)**. BDNF immunoreactivity was restricted to the blood vessels and the meninges (arrows in **A**), which remained unchanged according to data of real-time PCR using templates from whole-brain RNAs **(C)**. Scale bar: 20 μm. ns: not significant (Mann–Whitney).

**FIGURE 6:**
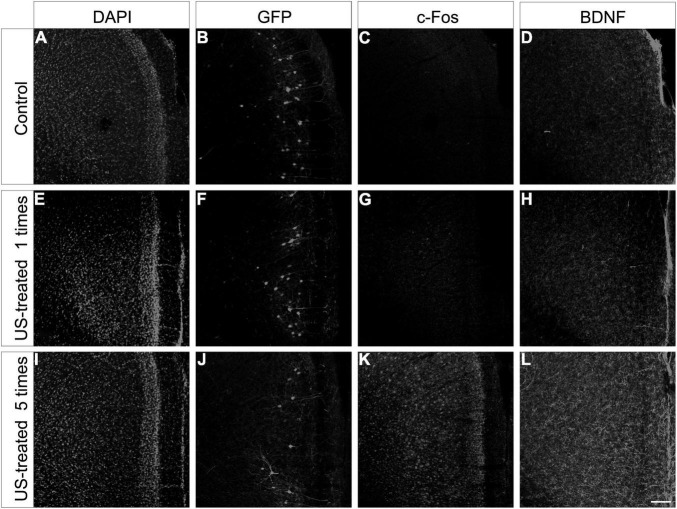
Repeated ultrasound stimuli elevate c-Fos and BDNF expression in the cortex. Confocal images showing the retrosplenial cortex from P30 mice sampled from the non-treated control **(A–D)**, the US-stimulated at E18.5 group **(E–H)**, and the US-stimulated five times group **(I–L)**. Each section was multiplied stained, and the histochemical reactions were separated with different fluorescent color channels and illustrated in grayscale. The GFP labeling was carried out at E14.5, and the samples show the mature pyramidal cells of the retrosplenial cortex from the non-stimulated control **(B)**, the US-stimulated at E18.5 group **(F)**, and the case of repetitive (once per week) US-stimulated group **(J)**. The c-Fos **(C,G)** and BDNF **(D,H)** immunoreactivities were similar between the control and the single US-stimulated group. A marked increase in c-Fos and BDNF immunopositivity could be observed in the 5 times US-stimulated group **(K,L)** compared to the non-stimulated control sample **(C,D)**. Sections were also stained with DAPI **(A,E,I)** for visualizing better the cortical layers. Scale bar: 100 μm.

**FIGURE 7:**
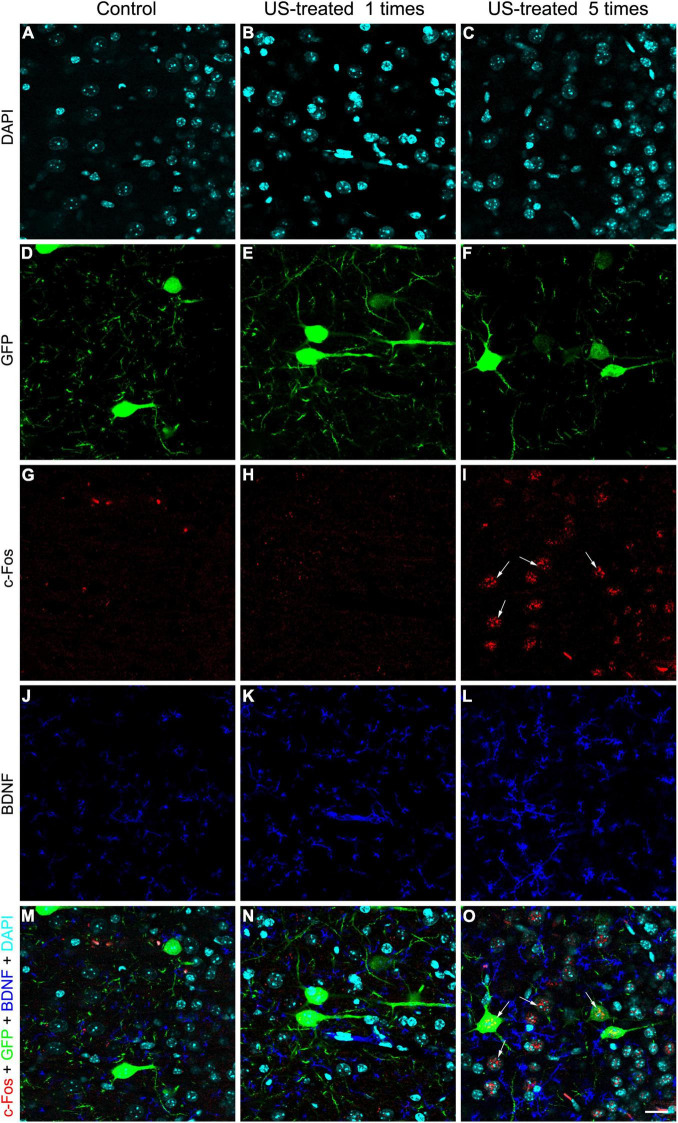
Weekly repeated ultrasound stimuli elevated c-Fos and BDNF expression in the cortex. Single-plane (0.51-μm-thick) confocal images showing an area with higher magnification of the retrosplenial cortex are shown in Figure 6 focusing on the layer 5 GFP-labeled pyramidal neurons **(D–F)**, c-Fos **(G–I)**, and BDNF **(J–L)**; immunohistochemistry and DAPI staining **(A–C)** in separated fluorescent color channels and merged colors **(M–O)**. Faint immunopositivity of BDNF and c-Fos was observed in the non-stimulated control and the single US-stimulated samples **(J,K,G,H)**. Visible c-Fos immunoreactivity was observed in the group which was stimulated with US 5 times (**I**, arrows) in GFP-labeled neurons and in other cells **(O)**, and the BDNF immunoreactivity was also increased **(L)**. Scale bar: 20 μm.

To test these hypotheses, we investigated the expression of c-Fos after a single US stimulation at E18.5 (Figure 5). The expression of c-Fos did not show any notable immunofluorescent reaction in the non-stimulated control brain (Figure 5A), but the immunoreactive signal was markedly increased due to the US stimulation (Figure 5B). This transient c-Fos signal returned to the base level 30 days after the stimulation (Figures 6G, 7H) compared to the non-stimulated control mice (Figures 6C, 7G). Repeated stimulation switched on the c-Fos expression virtually in all cell nuclei in the cortex (Figures 6A,C,E,G,I,K, 7A–C,G–I), which were not restricted to the GFP labelled neurons (Figures 7A–F,M–O). The BDNF immunoreactivity at E18.5 was mostly restricted to the meninges and blood vessels (Figure 5A). In contrast to c-Fos, a single US stimulation did not change the immunoreactivity of the BDNF in the cortex (Figures 5A,B); also, the BDNF at the mRNA level did not show a significant difference after 60 min of the stimulation (Figure 5C). In contrast to this, repetitive US stimulation increased the BDNF immunoreactivity in the retrosplenial cortex (Figures 6D,H,L, 7J–L).

Intrauterine US stimulation can also alter the migratory properties of immature cortical neurons (Ang et al., 2006), but neither a single nor repetitive US stimulation resulted in any visible changes in the layered distribution and basic morphology of the layer 5 pyramidal cells (Figures 6B,F,J) in our experiments. We considered that the lack of this phenomenon was due to the termination of migration of layer 5 neurons by the time the first US stimulation started (at E18.5).

### GFP Positive Pyramidal Neurons Are Positive for TRPC4

**FIGURE 8:**
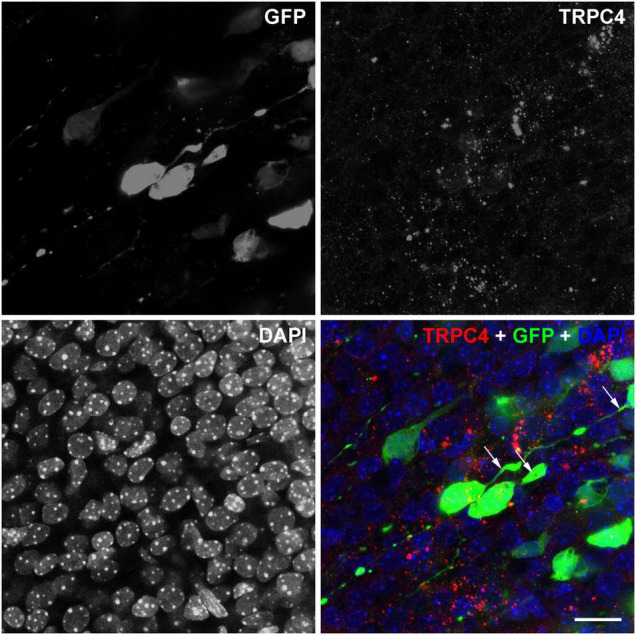
TRPC4 is expressed in GPF-labeled pyramidal cells. Confocal stacks from the E18.5 cortex in separated and merged fluorescent color channels show immature neurons labeled with GPF (green) at E14.5. The nuclei of the cells were labeled with DAPI (blue). TRPC4 immunoreactivity (red) showed a dot-like pattern in the cytoplasm and some cases also in the (leading) processes of the GFP labeled neurons (arrows). Scale bar: 20 μm.

Since the adjustments of the parameters, diagnostic HFU is lower than the threshold for the cavitation of the cell membrane, which can elevate directly the c-Fos levels in the cells (Grembowicz et al., 1999); we hypothesize receptor-mediated signaling behind the elevated c-Fos after the US stimulation. To understand better the possible signaling mechanisms of US-stimulus-dependent c-Fos activation, we focused on the expression of mechanosensitive receptors at E18.5. The TRPC4 immunoreactivity was distributed in a puncta-like manner in the GFP-labeled (at E14.5) pyramidal cells in the presumptive retrosplenial cortex at E18.5 (Figure 8). We also found TRPC4-immunopositive signals in the GFP-labeled pyramidal cell processes as well where the presence of the endoplasmic reticulum was questioned. Since the GFP-labeled neurons were positive for TRPC4, there was a possibility that US stimulus elevated the Ca^2+^ concentration in the cytoplasm of these neurons.

## Discussion

Our results demonstrate that even a single short-term diagnostic US has small but relatively long-lasting consequences on neuronal morphology through increasing the numbers of basal dendrites of layer V pyramidal cells in the retrosplenial cortex, a limbic area of the brain. The limbic area is involved in many functions in the limbic system including learning and navigation in rodents (for review, see Vann et al., 2009). A single US exposure did not but repetitive US exposure resulted in obvious biochemical changes in neurons marked by the increased expression of the early response gene c-fos and one of the secreted morphogenetic molecules BDNF.

### Neuronal Differentiation Under Ultrasound Exposure

The 14th embryonic day in mouse cortical development is corresponding to the event of the 12th week of the human cortical developmental stage (the birth of layer V neurons), the applied US exposure at E18.5 in mouse effect on all neurons in the nervous system similarly to US exposure in the human pregnancy after the 30th week (van den Ameele et al., 2014). Pre- and perinatal US exposure can regulate many aspects of brain development, particularly neuronal migration (Ang et al., 2006). A 30-min-long, 6.7-MHz pulsed US stimulation slowed down the migration of neurons populating the neocortex, resulting in some pyramidal cells settling in the improper layer or even stuck in the white matter (Ang et al., 2006). Since we applied the US stimulation at E18.5 at the time when the layer V neurons have arrived at their destination, studying the possible effect on neuronal migration in the developing limbic areas was not relevant in our experimental circumstances. However, we cannot exclude the effect of the 3-MHz frequency US exposure used in our study on neuronal and glial cell migration. Migrating pyramidal neurons are already polarized cells with the leading process that differentiate into the apical dendrite while the axon develops from the trailing process (Miller, 1988; Hatanaka and Murakami, 2002; Hatanaka et al., 2012). Our data showed that US exposure increased the number of the basal dendrites in the layer V pyramidal cells, which were newly formed instead of differentiation of existing migratory processes. This is because dendritic arbor develops in a highly orchestrated manner, which is quite a dynamic process that requires cytoskeletal rearrangements resulting in continuous growth and withdrawal movements of a newly formed dendritic process responding to electrical, microenvironmental, or intrinsic signals (Wong and Wong, 2000).

### The Role of Mechanosensitivity in the Growth of Neuronal Processes

The exact mechanisms of how the US can modulate the growths of neuronal processes during neuronal polarization and differentiation remain to be answered. Mechanical stimulation (tension) *in vitro* promotes neural stem cell differentiation and makes their dendritic arbor more extensive (Ang et al., 2006; Franze, 2013). One other possible reason for the higher number of dendrites in our experiments was that growing neurites could be destabilized by US stimuli and cause cytoskeletal rearrangements. It has been reported that rapid cytoskeletal reorganization after US exposure (0.5 MHz for 10 min) resulted in shorter primary cilia on hippocampal CA1 neurons from the rat *in vitro* slices, while US exposure stabilized those of dendrites and axons whose growth was terminated and synaptic contacts were formed (Franze, 2013; Hu et al., 2013; Huang et al., 2019). Mechanical tension can be translated to molecular signalization at a cellular level. A large set of mechanosensitive channels was reported to be able to respond to US including Piezo1, TRPA1, TRPC4, TRPV4, MEC-4, two-pore domain potassium channels (K2Ps), or voltage-gated sodium and Ca^2+^ channels (Kubanek et al., 2016, 2018; Prieto et al., 2018; Ye et al., 2018; Oh et al., 2019; Shen et al., 2020; Sorum et al., 2021). How US-mediated activation of these ion channels translated to dendritic growth is not exactly known. Nevertheless, US exposure facilitates this process *via* MAPK or ERK1/2 pathways due to the increased intracellular Ca^2+^ level through mechanosensitive ion channels like TRPV4 and Piezo1 (Shen et al., 2020). The expression level of most of these channels in the embryonic mouse brain is quite low or even negative except for TRPC4. TRPC4 starts to express at E13.5 dominantly in the subpallial regions, and the expression pattern expands to the cortex including the limbic area at the age of E18.5 and the TRPC4 is expressed by the cortical neurons throughout adult life (Figure 8; Fowler et al., 2007; Sunkin et al., 2013). Since we applied the US exposure at E18.5 or later, it was a great chance that this stimulus activated the TRPC4 channels. TRPC4 mechanosensitive ion channels play an important role in the mechanism of US stimulation and regulate the neurite outgrowth possibly through increasing Ca^2+^ efflux from the endoplasmic reticulum (Sun et al., 2014) and activating CaM kinase and CREB-mediated pathways (Redmond et al., 2002). Animals missing TRPC4 in channels had significantly reduced sensitivity to US stimulation; they consistently found larger wild-type responses to US. In contrast, animals lacking functional TRPC4 had reduced large reversal responses to US stimulation (Sun et al., 2014; Ibsen et al., 2015).

### Ultrasound Stimuli Activate Early Response Genes and the Release of Neurotrophins

The activation of neuronal mechanosensitive receptors and ion channels by US exposure can activate a signaling cascade that results in increased expression of c-jun and c-fos. Figure 5 shows that a single 3-MHz 10-min-long US exposure elevated the c-fos immunoreactivity in the fetal mouse brain an hour after the stimulation. A prompt and transient expression of c-fos regulates many aspects of circuit behavior including learning and memory (Tischmeyer and Grimm, 1999; Guzowski, 2002; Kubik et al., 2007). The c-fos activation upon BDNF release in the rat retrosplenial cortex required for fear-motivated long-term memory indicated that BDNF acted as an upstream signal for c-fos and increased BDNF expression in the brain elevated the c-fos expression (Katche and Medina, 2017). In contrast with this, we could not confirm the increase of BDNF mRNA and protein after 1 h of the US stimulation in E18.5 mice (Figure 5), but repetitive US stimulation greatly increased the expression of both BDNF and c-fos (Figure 6). This finding suggested that US stimuli achieved the increase in c-fos levels through mechanosensitive receptor-mediated signaling pathways (Cui et al., 2019; Qiu et al., 2019, 2020) that can lead to the elevation of the BDNF expression level acting downstream for the c-fos activation (Lee et al., 1997). Nevertheless, there was also a possibility that BDNF was released by storage from blood vessels or other sources (Nakahashi et al., 2000; Fujimura et al., 2002). BDNF has a pivotal role during dendritic arborization, promoting the elaboration of the dendritic tree, while it is not required for the proliferation, survival, or migration (Gorski et al., 2003; Jan and Jan, 2010). BDNF activates the PI3K-mTOR and MAPK pathways, which regulate the dendritic tree complexity and dendritic pattern; additionally, the PI3K-mTOR signaling modifies the expression of Reelin, which also has a pivotal role in the branching of hippocampal neurons (Dijkhuizen and Ghosh, 2005; Kumar et al., 2005; Jossin and Goffinet, 2007).

### Ultrasound Stimuli Alter the Activity and Morphology of Neuronal Circuits

US exposure alters neuronal activity in a frequency-dependent manner (Dong et al., 2006; Tufail et al., 2010; Yang et al., 2015; Hatch et al., 2016; Liu et al., 2017; Miller et al., 2017), which can have long-lasting effects on the activity of neurons that result in morphological changes at the synaptic and dendritic levels. The alterations of dendritic branching and pattern modify the local neural circuits and may cause changes in behavior and cognitive functions; earlier studies showed that a low dose (4 min, 3.5 MHz, MI: 0.1, TI: 0.1) of US exposure may enhance cognitive functions of rat pups according to the Morris water test, but using a higher dose (20 min, 3.5 MHz, MI: 1.4, TI: 1.0) of US leads to the opposite result (Li et al., 2015). The changing of mechanical properties of US stimulation can cause different effects also on neural cells *in vivo* (Tsui et al., 2005). Similar results can be observed with mouse pups, whereas a significant difference was observed related to the prenatal US in memory and cognitive function (US exposure was at E14.5); however, postnatal growth and physiological features were the same in the treated and control groups (Devi et al., 1995).

### Limitations

Since dendritic development is a lifelong process, the presented data here correspond to the young and immature nervous system only. Also, we cannot exclude the possibility that the surgical procedure, the electroporation, or even the GFP expression can alter the susceptibility of the neuron to US and masked our measured morphometric data.

## Conclusion

The worldwide applied physical parameters of fetal screening US exposure *in vivo* influence the number of basal dendrites of TRPC4 positive cortical pyramidal cell after only one US exposure; additionally, repetitive US elevates the level of BDNF and c-Fos proteins indexing a possible molecular background of this morphological changing. Since US stimuli cause mild morphological changes in developing brain circuits, it can be an exciting tool for regenerative medicine for those with neurodevelopmental disorders accompanied by a decrease in the number of dendrites.

## Data Availability Statement

The original contributions presented in the study are included in the article/supplementary material, further inquiries can be directed to the corresponding author/s.

## Ethics Statement

The animal study was reviewed and approved by the Committee for Animal Research Studies at the University of Debrecen.

## Author Contributions

TP designed and carried out the US stimulation protocols, analyzed the data, wrote the manuscript, and led the project. ZF, EK, and BS performed the neuron reconstruction, analyzed the data, and wrote the manuscript. AV designed and constructed the expression vectors. MP designed the artworks and reviewed the manuscript. EB conceived the idea and critically reviewed the manuscript. GO and GH performed the qPCR experiments and wrote the analysis and reviewed the manuscript. PS analyzed and wrote the morphometric data. ZM performed the *in utero* electroporation and immunohistochemistry, conceived the research, wrote the manuscript, and led the project. All authors contributed to the article and approved the submitted version.

## Conflict of Interest

The authors declare that the research was conducted in the absence of any commercial or financial relationships that could be construed as a potential conflict of interest.

## Publisher’s Note

All claims expressed in this article are solely those of the authors and do not necessarily represent those of their affiliated organizations, or those of the publisher, the editors and the reviewers. Any product that may be evaluated in this article, or claim that may be made by its manufacturer, is not guaranteed or endorsed by the publisher.
